# Duodenal stenosis associated with an ectopic opening of the common bile duct into the duodenal bulb: a case report

**DOI:** 10.1186/s40792-021-01351-z

**Published:** 2021-12-16

**Authors:** Kazuki Kobayashi, Michinori Murayama, Hidekazu Sugasawa, Makoto Nishikawa, Kiyoshi Nishiyama, Hiroaki Takeo

**Affiliations:** 1 Department of Surgery, Self-Defense Forces Central Hospital, 1-2-24 Ikejiri, Setagaya, 154-0001 Japan; 2 Department of Diagnostic Pathology, Self-Defense Forces Central Hospital, 1-2-24 Ikejiri, Setagaya, 154-0001 Japan

**Keywords:** Ectopic opening of the common bile duct, Duodenal ulcer, Duodenal stenosis, Groove pancreatitis

## Abstract

**Background:**

Ectopic opening of the common bile duct is a rare congenital biliary anomaly. Herein, we present a case of duodenal stenosis with ectopic opening of the common bile duct into the duodenal bulb.

**Case presentation:**

A 54-year-old man was referred with fever, nausea, and vomiting. He had experienced epigastric pain several times over the past 30 years. Endoscopy showed a post-bulbar ulcer, a submucosal tumor of the duodenum, and a small opening with bile secretion. Contrast duodenography revealed duodenal stenosis and bile reflux with a common bile duct deformity. Pancreatoduodenectomy was performed because of the clinical suspicion of a biliary neoplasm or groove pancreatitis. The resected specimen showed an ectopic opening of the common bile duct into the duodenal bulb and no tumor.

**Conclusions:**

Ectopic opening of the common bile duct into the duodenal bulb is complicated by a duodenal ulcer, deformity, and stenosis mimicking groove pancreatitis or pancreatic tumors. Although rare, we should be aware of this anomaly for an accurate diagnosis.

## Background

Various opening sites have been described in the literature of the ectopic opening of the common bile duct (EOCBD), such as the 3rd or 4th portion of the duodenum, pyloric canal, duodenal bulb, and stomach. Some cases of EOCBD in the duodenal bulb develop complications such as duodenal ulcers, deformities, biliary stones, tumors, and stenosis [1–19]. Herein, we present a case of duodenal stenosis with EOCBD in the duodenal bulb.

## Case presentation

**Fig. 1:**
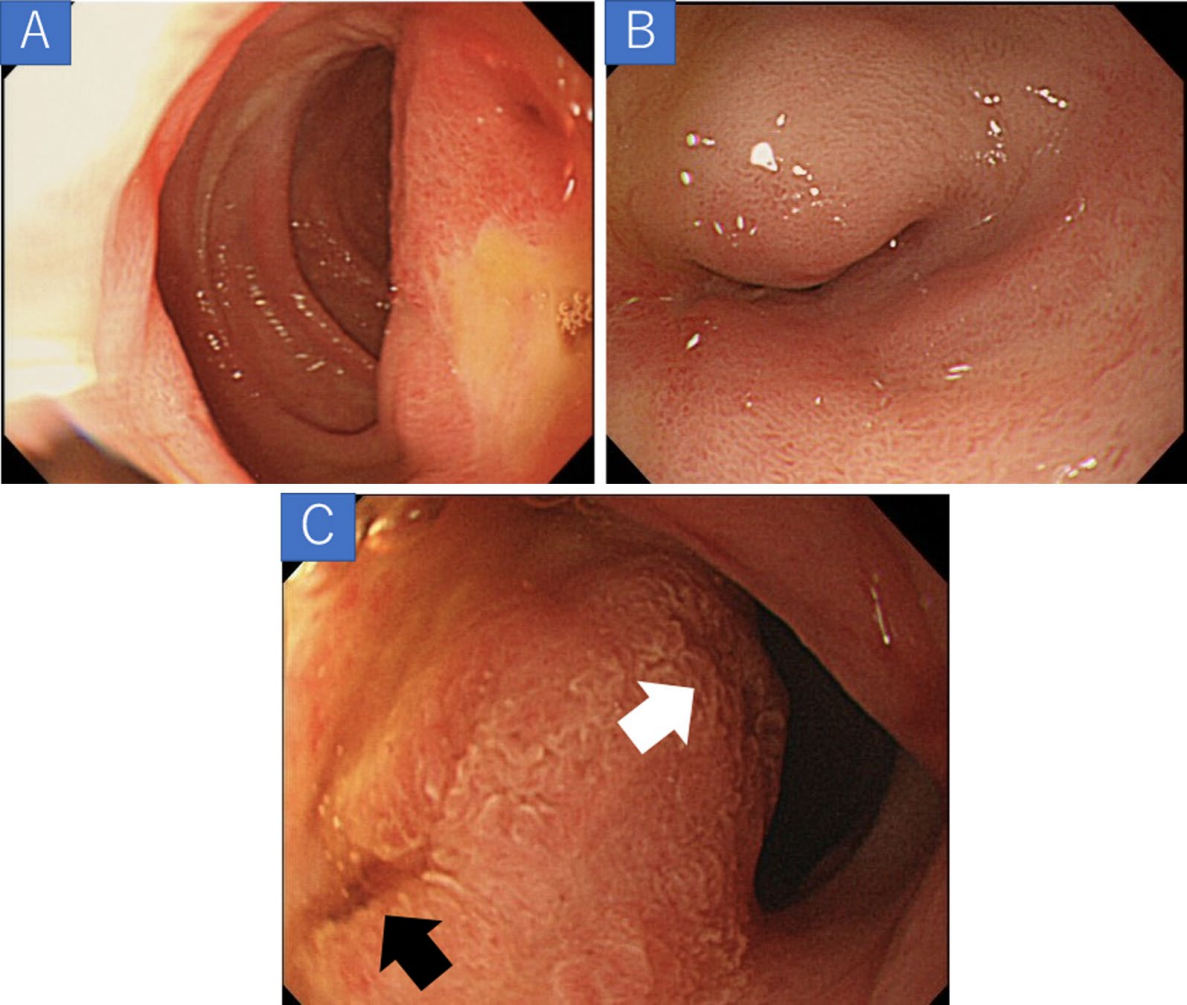
Endoscopic photograph. **A** The active duodenal ulcer, proximal to the duodenal stenosis. **B** The duodenal submucosal tumor at the duodenal bulb. **C** The papilla of Vater (white arrow) and the small opening with bile excretion (black arrow)

**Fig. 2:**
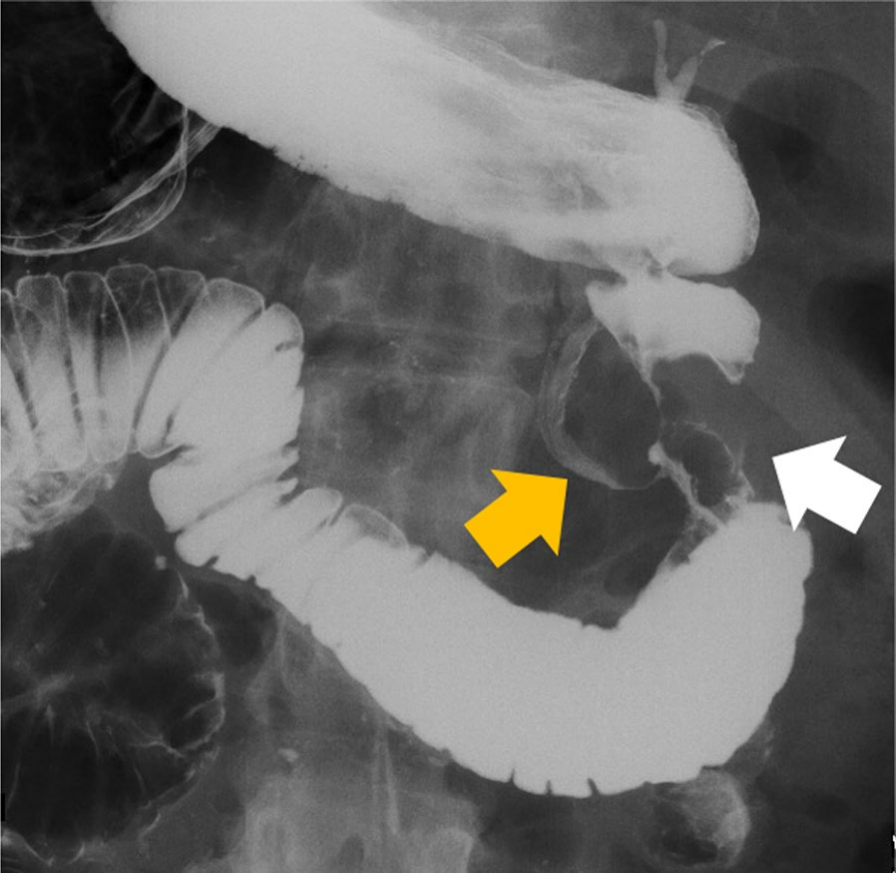
Contrast duodenography image. Duodenal stenosis (white arrow) and bile duct reflux (orange arrow) with a hook-like appearance

**Fig. 3:**
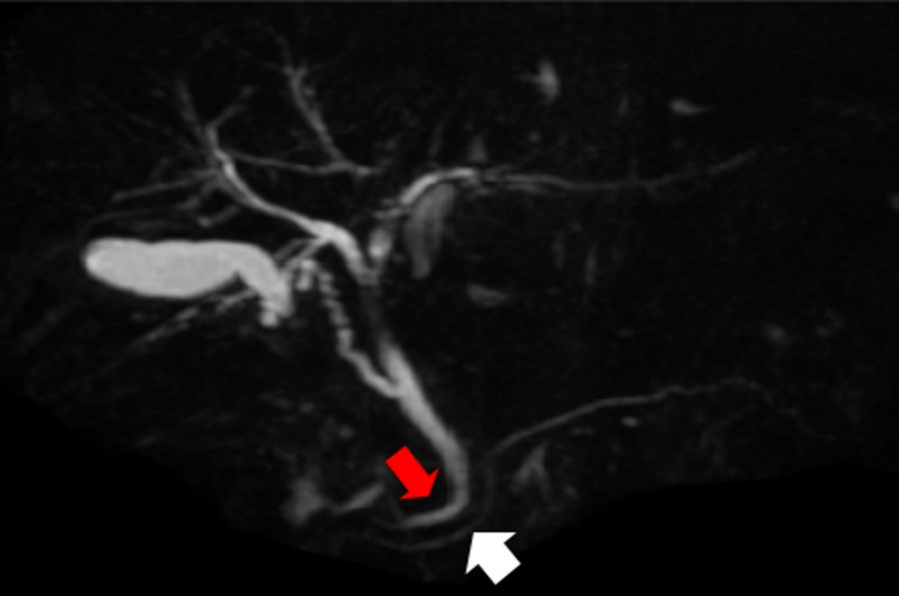
Magnetic resonance cholangiopancreatography image. The CBD (red arrow) exhibits a hook-like configuration. Separate opening between the CBD and pancreatic duct (white arrow)

**Fig. 4:**
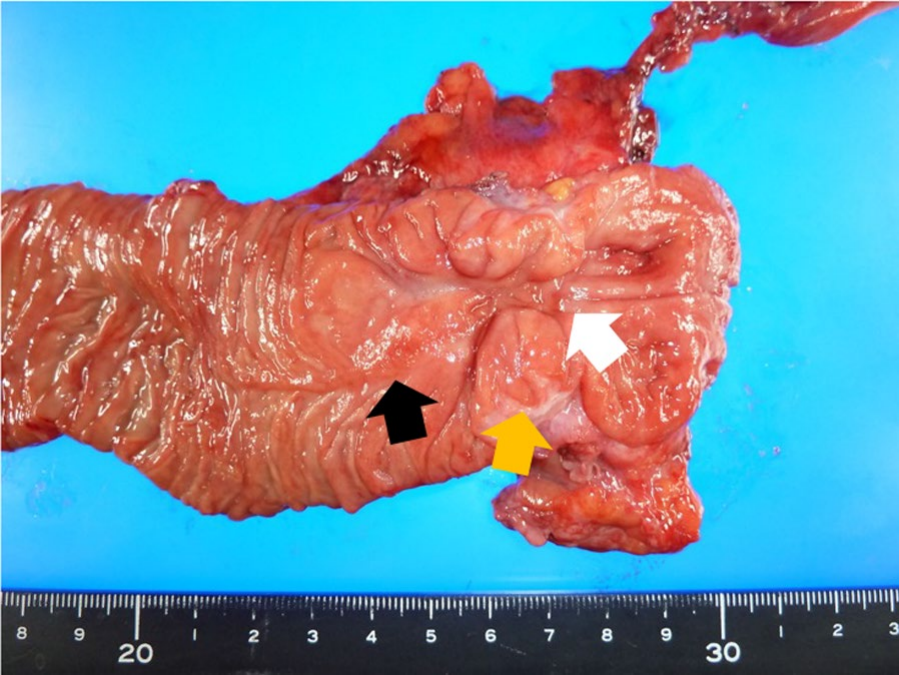
Macroscopic examination of resected duodenum. Duodenal stenosis (white arrow), CBD opening (orange arrow), and papilla of Vater (black arrow)

**Fig. 5:**
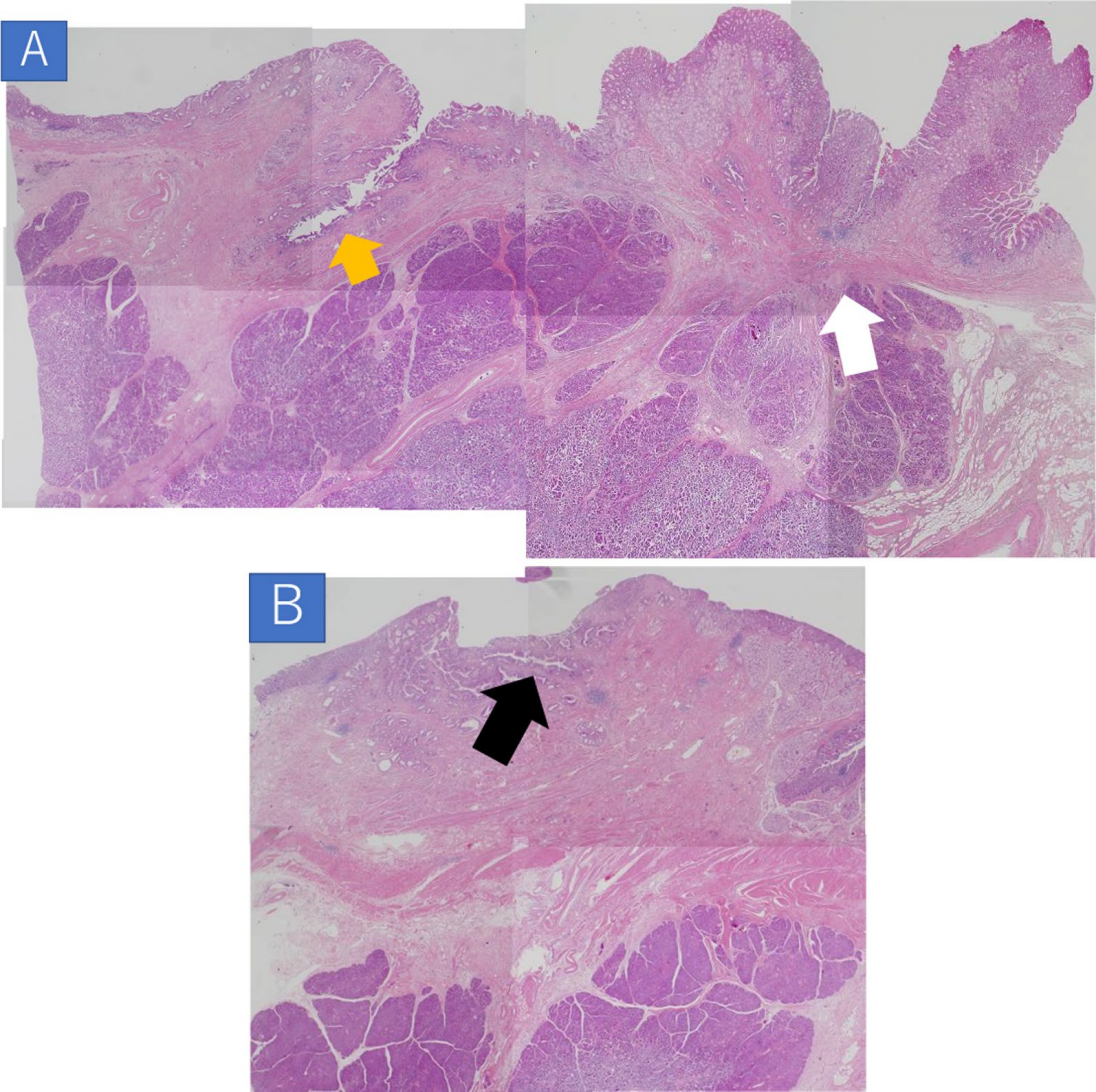
Histopathological findings. **A**, **B** The CBD and pancreatic duct were separate openings with epithelial cells. **A** Duodenal stenosis: fibrosis without tumor cells (white arrow). Opening of the CBD: no sphincter muscle structure (orange arrow). H&E stain, × 20. **B** The papilla of Vater: separate opening of the pancreatic duct (black arrow) with only the muscle structure. H&E stain, × 2

A 54-year-old man visited a local clinic with complaints of fever, nausea, and vomiting. Laboratory studies revealed elevated serum aspartate aminotransferase of 42 IU/L, serum alanine transferase of 199 IU/L, serum γ-glutamyl transpeptidase of 602 IU/L, and serum lipase of 61 U/L. Endoscopy revealed a duodenal submucosal tumor. The patient was referred to our hospital for further investigation. He had a gastroduodenal ulcer and experienced occasional epigastric pain for the past 30 years. He received treatment for eradication of *Helicobacter pylori* at 43 years of age. Endoscopy at our hospital revealed stenosis of the duodenal bulb, which was suspected to be caused by a submucosal tumor and an active ulcer (Fig. 1A and B). In addition, bile excretion was only observed in a small opening near the ulcer (Fig. 1C). Abdominal computed tomography (CT) revealed normal findings in the duodenum, but gas in the bile ducts (pneumobilia). Contrast duodenography showed duodenal stenosis and reflux of the bile with a bile duct deformity (Fig. 2). Magnetic resonance cholangiopancreatography (MRCP) showed severe angulation of the CBD (appearing as a hook-shaped configuration), which opened at a different site from the pancreatic duct (Fig. 3). Endoscopic ultrasonography revealed hypoechoic lesion in the 2nd portion of the duodenum which was originating from the muscularis propria. It was difficult to distinguish inflammatory thickening from submucosal tumor. Endoscopic ultrasound fine-needle aspiration revealed inflammatory cells, and no evidence of neoplastic cells. Positron emission tomography CT showed [18F]-fluorodeoxyglucose (FDG) accumulation in the 2nd portion of the duodenum (standard uptake value—MAX: 4.15), but the delayed phase showed a decrease in accumulation. Based on these test results, a suspected diagnosis of groove pancreatitis was made, but with the findings of duodenal stenosis and hook-shaped CBD, the possibility of biliary neoplasm complicating choledochoduodenal fistula was also entertained. There was the possibility of malignancy; and surgical management was considered the rational treatment for the symptomatic groove pancreatitis, so we decided to perform pancreaticoduodenectomy. The patient’s complaints were relieved, and the laboratory data improved after surgery. The resected specimen revealed the papilla of Vater located in the 2nd portion of the duodenum, and the CBD opened approximately 2 cm proximal to the papilla of Vater (Fig. 4). Histologically, the CBD opening had no sphincter muscles (Fig. 5A). The duodenum proximal to the opening of the CBD was stenotic due to ulceration and fibrosis. The CBD and pancreatic duct were separate openings with epithelial cells (Fig. 5A and B). These findings correlated with the diagnosis of EOCBD into the duodenal bulb.

## Discussion

Here, we have reported a case of duodenal stenosis with EOCBD. The true incidence of patients with an EOCBD into the duodenal bulb is unclear. Sezgin et al. [11] reported that 1.05% of patients undergoing endoscopic retrograde cholangiopancreatography (ERCP) had an EOCBD at various sites of the upper digestive tract, such as the pyloric canal, stomach, and duodenal bulb. In a previous study by Lee et al. 18 out of 16,541 patients who underwent ERCP had an EOCBD into the duodenal bulb [4]. Taş et al. reported that 20 out of 3270 patients who underwent ERCP had an EOCBD into the duodenal bulb (0.61%) [8].

As in our case, EOCBD patients are usually elderly men. Erkan et al. reported that 49 out of the 53 patients were male. Lee et al. and Lee et al. reported that 7 out of 8 and 15 out of 18 patients were males, respectively [3, 4].

The cases of EOCBD into the duodenal bulb have characteristic clinical features. One such feature is duodenal ulcer. Lee et al. [4] and Taş et al. [18] reported that 13 out of 18 patients (72%) and 16 out of 20 patients (80%) had a history of duodenal ulcers, respectively. Duodenal ulcers may be associated with apical stenosis or deformity. In a previous study, 13 out of 18 patients with EOCBD had active duodenal ulcers, and 9 out of 13 patients had duodenal deformity [4].

The cause of the association between duodenal deformity and ulcers is not clearly known. Lee et al. associated the increased incidence of duodenal ulcer and deformity with the constant exposure of the duodenal bulb to bile acid [3]. Bile reflux into the duodenal bulb causes an increase in pH. At high pH, bile acids may cause gastric and duodenal bulb mucosal damage [7, 8, 11, 12, 15]. Previous reports also suggest that bicarbonate in pancreatic secretion contributes to the increase in the pH in the bulb, leading to recurrent duodenal ulcer and deformity [12]. The presence of pancreatic secretion, in addition to bile, seems to be necessary for the development of deformity, ulcers, and stenosis.

Another feature is recurrent cholangitis, with or without stones. Reflux cholangitis may occur due to the absence of the sphincter muscles. These patients are often treated surgically or endoscopically. During endoscopic balloon dilation, satisfactory placement of a balloon catheter through an ectopic opening is difficult because of the angulated distal CBD and the high risk of perforation or bleeding [11, 12, 19]. Therefore, surgical treatment is a better alternative. This characteristic angulation of the distal CBD is called a “hook-like” appearance [3]. In our case, the CBD showed the “hook-like” deformity. Table 1 shows the past reported series of bile duct anomaly with the occurrence rate, duodenal ulcer rate, cholangitis rate, and treatment approaches [3–9, 11, 12, 14, 15, 17–20].

**Table 1:** The past report of EOCBD into the duodenal bulb

No.	Author	Year	Number of cases	Duodenal ulcer (*n*)	Duodenal stenosis (*n*)	Cholangitis (*n*)	Treatment					References
							Surgical treatment			Endoscopic treatment (*n*)	Medical or no treatment (*n*)	
							BB (*n*)	PD (*n*)	Others (*n*)			
1	Lee et al	1997	8	7	–	5	6	1			1	[3]
2	Lee et al	2003	18	13	9	7	12	1		4	1	[4]
3	Krstic et al	2005	1	0	1	1	1					[7]
4	Disibeyaz et al	2007	53	8	26	23			8	28	17	[8]
5	Song et al	2007	1	1	0	1	1					[11]
6	Sezgin et al	2010	10	2	–	3	4		1	3	2	[20]
7	Salitas et al	2010	9	7	–	–	3			3	3	[12]
8	Parlak et al	2010	74	–	–	30			13	60	1	[5]
9	Üsküdar et al	2012	1	0	1	1				1		[6]
10	Lee et al	2015	1	0	0	1	1					[9]
11	Takikawa et al	2016	1	0	0	1				1		[14]
12	Taş et al	2018	20	16	16	12			14	6		[15]
13	Lee et al	2019	1	1	0	0					1	[17]
14	Peng et al	2019	8	3	8	3	–					[18]
15	Muhammedoǧlu et al.	2019	2	0	0	2	1			1		[19]
Total, *n* (%)			208	58 (43.2)	61 (57)	90 (45.2)	29 (14.5)	2 (1)	36 (18)	107 (53.5)	26 (13)	

*BB* bilioenteric bypass, *PD* pancreaticoduodenectomy, – not mentioned about it

In our case, one of the differential diagnoses was groove pancreatitis. Lee et al. reported a case of EOCBD accompanied by groove pancreatitis [9]. Groove pancreatitis is a segmental chronic pancreatitis that affects the anatomical area between the pancreatic head, duodenum, and common bile duct, which is referred to as the groove area [20]. Conservative treatment or endoscopic drainage of the minor papilla is effective for treating groove pancreatitis [9, 21]. However, surgical procedures, such as pancreaticoduodenectomy or pylorus-preserving pancreaticoduodenectomy, are the treatment of choice when the symptoms do not improve, or when the differential diagnosis includes pancreatic carcinoma [20, 22–25]. Pezzilli et al. [22] and Sanada et al. [23] reported that 72% and 55% of patients underwent surgery, respectively, for such reasons. Iemoto et al. [24] reported a case that underwent pancreaticoduodenectomy for groove pancreatitis associated with a duodenal ulcer, similar to our case.

## Conclusions

EOCBD into the duodenal bulb may be associated with biliary and duodenal diseases, such as recurrent duodenal ulcers. Therefore, EOCBD into the duodenal bulb should be considered in patients with recurrent duodenal ulcers and duodenal stenosis.

## Data Availability

The datasets used and/or analyzed during the current study are available from the corresponding author upon reasonable request.

## Abbreviations

EOCBD: Ectopic opening of common bile duct
CT: Computed tomography
ERCP: Endoscopic retrograde cholangiopancreatography
FDG: Fluorodeoxyglucose
BB: Bilioenteric bypass
PD: Pancreaticoduodenectomy
